# Whole-Genome Sequence Analysis of an Endophytic Fungus *Alternaria* sp. SPS-2 and Its Biosynthetic Potential of Bioactive Secondary Metabolites

**DOI:** 10.3390/microorganisms10091789

**Published:** 2022-09-05

**Authors:** Jianyun Tao, Xuelian Bai, Mingyuan Zeng, Mengshi Li, Zhe Hu, Yunfen Hua, Huawei Zhang

**Affiliations:** 1 School of Pharmaceutical Sciences, Zhejiang University of Technology, Hangzhou 310014, China; 2 College of Life and Environmental Sciences, Hangzhou Normal University, Hangzhou 311121, China

**Keywords:** endophytic fungus, *Alternaria*, genome sequencing, antiSMASH analysis, GNPS, secondary metabolite

## Abstract

As one of the commonly isolated endophytic fungi, *Alternaria* has been known for the production of numerous secondary metabolites (SMs). However, its detailed genomic features and SM biosynthetic potential have not been extensively studied thus far. The present work focuses on the whole-genome sequencing and assembly of an endophytic strain *Alternaria* sp. SPS-2 derived from *Echrysantha chrysantha* Lindl. and gene annotation using various bioinformatic tools. The results of this study suggested that the genome of strain SPS-2 was 33.4 Mb in size with a GC content of 51% and an N50 scaffold of 2.6 Mb, and 9789 protein-coding genes, including 644 CAZyme-encoding genes, were discovered in strain SPS-2 through KEGG enrichment analysis. The antiSMASH results indicated that strain SPS-2 harbored 22 SM biosynthetic gene clusters (BGCs), 14 of which are cryptic and unknown. LS–MS/MS and GNPS-based analyses suggested that this endophytic fungus is a potential producer of bioactive SMs and merits further exploration and development.

## 1. Introduction

Endophytes have been recognized as one of the important sources of bioactive natural products with therapeutic potential [1,2]. *Alternaria* strains are commonly detected in plants and have been shown to be a treasure trove of secondary metabolites (SMs) since as many as 482 substances have been isolated and structurally elucidated from this genus by extensively searching the Dictionary of Natural Products database (accessed on 10 July 2022). Structurally, the SM inventory of the genus *Alternaria* consists of diverse groups, including terpenes, pyrones, cyclopeptides, nitrogen-containing compounds, and miscellaneous, which showed a variety of biological activities, such as antimicrobial effect, phytotoxicity, and cytotoxicity [3,4,5]. Additionally, these SMs are biosynthesized via an assembly line process that is catalyzed by modular polyketide synthases (PKSs), non-ribosomal peptide synthetase (NRPS), NRPS-PKS, terpene, and ribosomally synthesized and post-translationally modified peptides (RiPPs). Thus far, however, the detailed genomic features and SM biosynthetic potential of the *Alternaria* strain have been seldom reported.

In our continuous search for new SMs from endophytic fungi, an endophytic *Alternaria* strain SPS-2 was obtained from *Echrysantha chrysantha* Lindl., one of the traditional Chinese medicine used for the treatment of the swelling of the eye, ophthalmalgia, delacrimation, nephelium of the eye, and nocturnal emission [6,7]. To better understand this endophytic fungus and explore its SM biosynthetic potential, in this study, whole-genome sequencing and assembly were conducted by hybridizing next-generation sequencing (NGS) and using the Illumina MiSeq platform. Its gene annotation was extensively predicted using various BLAST databases, including non-redundant (Nr) protein sequence, Swiss-Prot, Gene Ontology (GO), Kyoto Encyclopedia of Genes and Genomes (KEGG) and EuKaryotic Orthologous Groups (KOG), and Carbohydrate-Active Enzyme (CAZy) databases. Additionally, an antibiotic and secondary metabolite analysis database (antiSMASH) was used to determine the SM biosynthetic potential of strain SPS-2.

## 2. Materials and Methods

### 2.1. Strain, Cultivation, and Crude Extracts Preparation

Endophytic strain SPS-2 was isolated and purified from leaves of the coastal plant *E. chrysantha* Lindl., which naturally grows on the bank of the intertidal zone of Hangzhou Bay (China). A suspension of culture containing mycelia in a potato dextrose agar (PDA) medium supplemented with glycerol (20% *v*/*v*) was stored at −80 °C in our lab at Zhejiang University of Technology (China).

Strain SPS-2 was cultured on PDA at 28 °C for 7 days. A balanced amount of fungal colony was transferred to culture broth in a 500 mL Erlenmeyer flask, which contained 250 mL potato dextrose broth (PDB) consisting of potato 200 g/L and glucose 20 g/L. This was followed by shaking at 200 rpm at 28 °C for 2 days that prepared it as seed broth; then, the seed broth was transferred to a fluid medium (glucose 20 g/L, maltose 20 g/L, mannitol 20 g/L, glutamate 10 g/L, peptone 5 g/L, and yeast extract 3 g/L) in a 1 L Erlenmeyer flask. After two weeks of incubation (200 rpm, 30 °C), all mediums (approximate 30 L) were extracted three times with the same volume of ethyl acetate (Fangping Chemical Co., Ltd., Hangzhou, China); the upper solvent was evaporated at 25 °C in a vacuum to yield extract (about 12.35 g).

### 2.2. Phylogenetic Analysis

Strain SPS-2 was inoculated into the PDA medium for a few days to culture and become cultivated, followed by morphological observations and the nuclear ribosomal internal transcribed spacer (ITS) gene amplicon sequencing. First, the sequence of ITS was amplified via polymerase chain reaction (PCR) with ITS1-ITS4 primer pairs. Additionally, 3 µL of the PCR product was taken for 1% agarose gel electrophoresis detection to confirm the PCR amplification fragment. Then, the products were recovered using an AxyPrep DNA Recovery Kit. Finally, sequencing was performed with an ABI3730-XL sequencer. Meanwhile, the ITS sequence with 1199 bp was submitted to GenBank in the NCBI database, and an accession number was acquired. A phylogenetic tree was constructed using MEGA (version 7.0, https://www.megasoftware.net/, accessed on 25 June 2022) by comparison with other *Alternaria* strains with high similarities in the NCBI database.

### 2.3. Genome Sequencing and Assembly

Strain SPS-2 was inoculated into the PDA medium and cultivated for three days at 28 °C. The fungal chromosome was extracted using the cetyltrimethylammonium bromide (CTAB) extraction protocol. The integrity and purity were assessed via 1% agarose gel electrophoresis, and then dissolved in sterile water and adjusted to a concentration of 149 ng/µL.

A total of 400 bp DNA fragment library was constructed using the whole-genome shotgun (WGS) method [8]. Sequencing was performed on the Illumina MiSeq platform and resulted in paired-end raw data in FASTQ format, followed by decontamination using Adapter Removal (version 1.5.4, Copenhagen, Denmark) by and quality correction using the SOAPec (version 2.0, https://help.rc.ufl.edu/doc/SOAPec, accessed on 3 February 2022) software [9]. The SOAPdenovo (version 2.04, http://soap.genomics.org.cn/soapdenovo.html, accessed on 10 February 2022) software was used to assemble and construct the scaffold sequence, while the GapCloser (http://soap.genomics.org.cn/soapdenovo.html, accessed on 20 February 2022) program was deployed to fill the sequence gap. Pilon (version 1.18, https://github.com/broadinstitute/pilon, accessed on 28 February 2022) was used to correct the splicing results and afford the final assembly [10]. Finally, an evaluation of the integrity of the predicted genomes was conducted using fungi_odb9 (fungal database) BUSCO (Benchmarking Universal Single-Copy Orthologs, version 3.0.2, http://busco.ezlab.org, accessed on 15 March 2022) [11].

### 2.4. Gene Prediction and Annotation

For gene prediction, Augustus (version 3.03, Göttingen, Lower Saxony Land, Germany), glimmerHMM (version 3.0.1, Baltimore, MD, USA), and SNAP (version 28 July 2006, Davis, CA, USA)were used to predict the gene model [12,13,14]. Then, Exonerate (version 2.2.0, https://www.ebi.ac.uk/about/vertebrate-genomics/software/, accessed on 20 March 2022) was used for homology prediction. Finally, EVidenceModeler (version 25 June 2012, Rockvill, MD, USA)was used to predict protein-coding genes by combining the models from the above software. The tRNA genes were predicted using tRNAscan-SE (version 1.3.1, http://lowelab.ucsc.edu/tRNAscan-SE/, accessed on 10 April 2022) and rRNA genes using RNAmmer 1.2 [15,16]. The prediction of non-coding RNAs was mainly achieved by comparison with Rfam [17].

Gene annotation is the functional analysis of all protein-coding genes, including predictions of information such as motifs, domains, protein functions, and metabolic pathways. Functional annotations on putative genes were carried out using the following bioinformatic tools: BLAST for Swiss-Prot and KOG databases (E-value threshold of 10^−6^), KAAS (version 2.1, Gokasho, Kyoto, Japan) for KEGG annotation [18], and InterPro (version 66.0, Hinxton, Cambridgeshire, UK) for GO annotation [19]. The Diamond (version 0.9.10.111, Tübingen, Germany) software was then used to run protein-coding genes against the NCBI nr database (E-value threshold of 10^−6^) [20].

### 2.5. Prediction of CAZymes

Carbohydrates contain a considerable amount of biological information and, therefore, are beneficial to analyze the metabolic process of strain SPS-2 and the differences between strains. The Hmmscan software (v3.1b2, http://hmmer.org/, accessed on 10 May 2022) was employed in prediction and annotation of the presence of carbohydrate-active enzyme (CAZyme)-related genes in strain SPS-2. Meanwhile, the data of 12 other *Alternaria* strains obtained from the CAZy database were compared with these of strain SPS-2 to further understand its carbohydrate degradation capacity.

### 2.6. Analysis of Secondary Metabolite Biosynthetic Gene Clusters

The SM biosynthetic gene clusters of strain SPS-2 were predicted using antiSMASH (version 6.1.1) (https://antismashdb.Secondarymetabolites.org/#/start, accessed on 30 June 2022) and further annotated using BlastP analysis.

### 2.7. Metabolomic Profiling of Strain SPS-2 Using LC–MS/MS

The LC–MS/MS analysis of crude extract of strain SPS-2 was conducted on a QTOF mass spectrometer (AB SCIEX X500B). A 1.8 μm Agilent ZORBAX Extend C18 (2.1 mm × 100 mm), maintained at 35 °C, was operated using a gradient elution of H_2_O and MeOH, running at 0.3 mL/min. The gradient program was as follows: 10% MeOH for 2 min, 10–100% MeOH for 16 min, and 100% MeOH for 5 min. Data dependent acquisition (DDA) mode in mass spectrometry was recorded in positive ion mode with a spray voltage of 5.5 kV and a temperature of 550 °C. Then, the raw LC–MS/MS data files were converted into an mzXML format using MSConvert, and the data were analyzed with MZ mine2 (version 2.53, https://github.com/mzmine/mzmine2/releases, accessed on 16 July 2022). Then, the data were uploaded to the Global Natural Products Social Molecular Networking (GNPS) web platform for molecular networking [21]. The Cytoscape software (version 3.9.1, https://cytoscape.org/, accessed on 16 July 2022)was used to visualize the resulting molecular networking, and known metabolites were annotated by comparing the mass and mass fragmentation pattern with GNPS spectral libraries [22].

## 3. Results and Discussion

### 3.1. Morphology and Phylogenetic Analysis of Strain SPS-2

**Figure 1:**
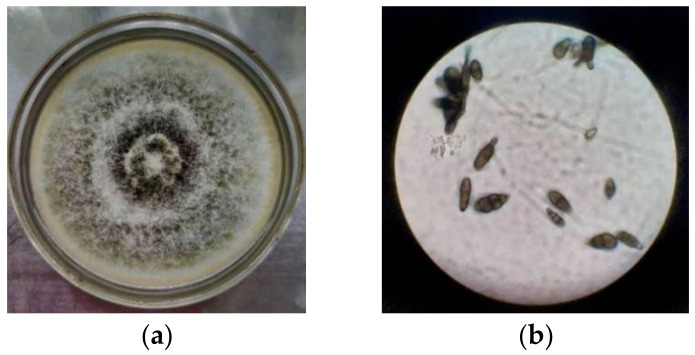
Colony (**a**) and spore (**b**) morphology of strain SPS-2.

**Figure 2:**
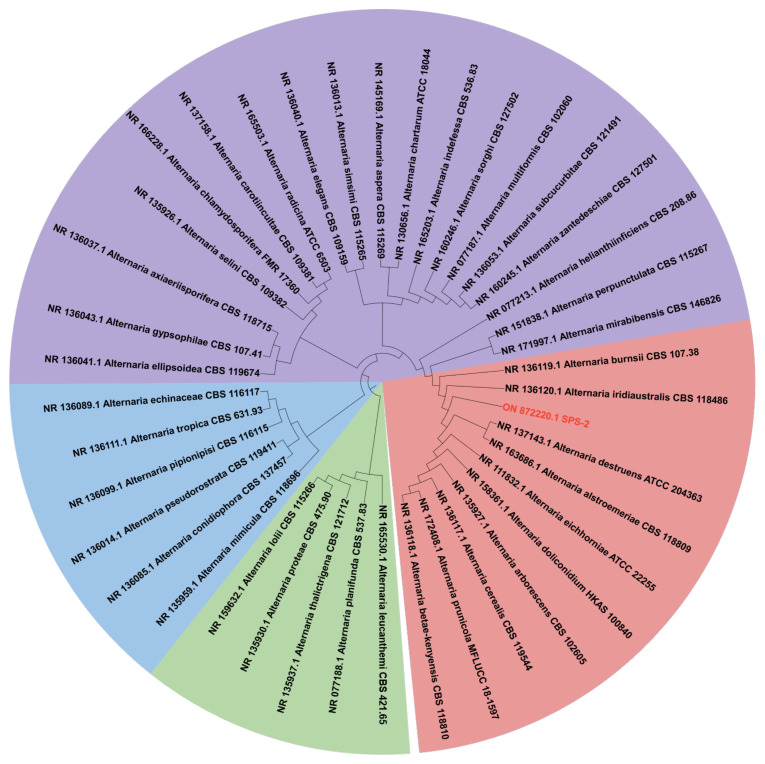
ITS-based phylogenetic tree of strain SPS-2 marked in red.

Spores of strain SPS-2 developed after culturing on a PDA medium for 6 days at 28 Global Natural Products Social Molecular Networking. Its white colonies grew rapidly on the PDA medium at the initial stage and darkened after 6 days (Figure 1). Subsequently, a phylogenetic tree was constructed for strain SPS-2 on the basis of its ITS sequence (GenBank accession no.: ON872220.1) and analysis, suggesting that this strain is the most closely related to the genus *Alternaria* (Figure 2).

### 3.2. Genome Sequencing and Assembly

The genome sequencing of strain SPS-2 afforded a sequence with a length of 33,400,178 bp with a G + C content of 51.0% and an N50 value of 2,610,814 bp (Table 1). Additionally, the integrity was 95.2%, indicating that the quality of the genome assembly was high (Table S1). The total CDS sequence length was 13,553,185 bp, accounting for 40.58% of the genome. Comparison with other *Alternaria* strains deposited in the NCBI database (Table 2) revealed that the genome size of strain SPS-2 was average, but it had the fewest gene-coding number. For non-coding RNA, 12 rRNAs,108 tRNAs, and 32 ncRNAs were predictably discovered in the genome of strain SPS-2 (Table 3). The number of repeating sequences was determined as 378,941 bp (1.13% of the genome), which was fewer than that of other *Alternaria* strains, such as *A. solani* (1.5%), *A. alternantherae* (16.5%), and *A. avenicola* (11.9%) [23]. The number of long terminal repeats was 26,157 bp, representing 0.08% of the whole genome, while the number of DNA elements was 12,782 bp, only occupying 0.04%.

### 3.3. Genome Annotation

**Figure 3:**
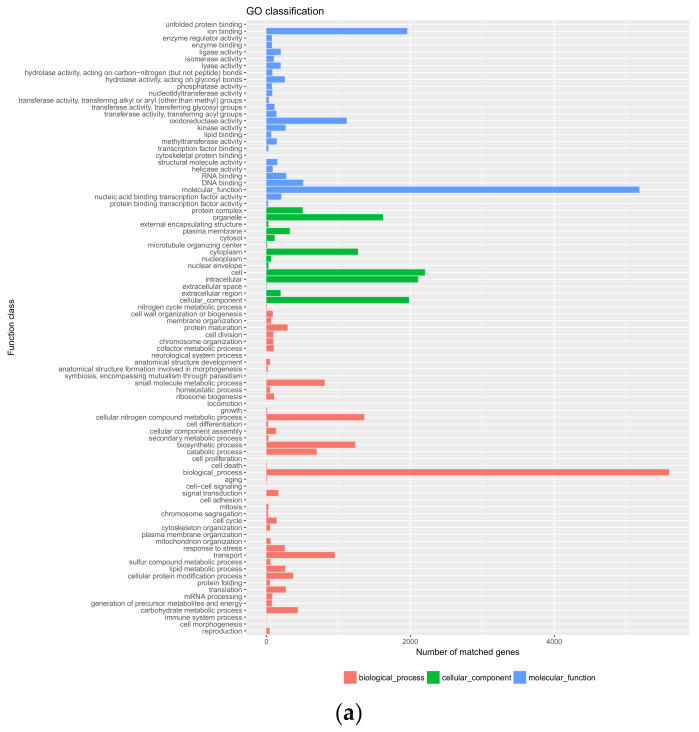

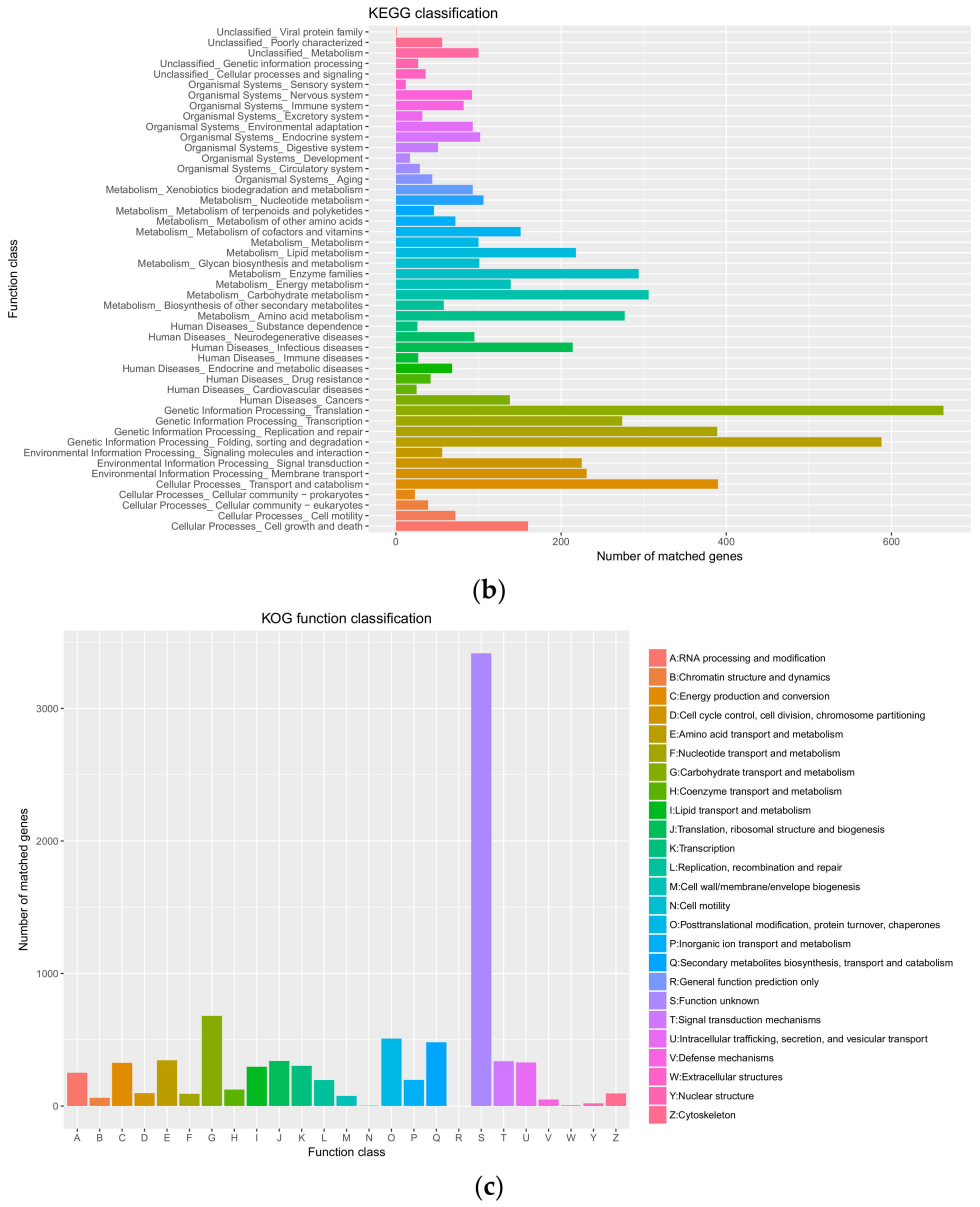
Functional annotation of strain SPS-2 genes encoding the proteins: (**a**) Gene Ontology (GO) analysis; (**b**) Kyoto Encyclopedia of Genes and Genomes (KEGG) analysis; (**c**) EuKaryotic Orthologous Group (GO).

To conduct the functional annotation of the gene model in strain SPS-2, the blast search function was used to enter the putative protein-coding sequences into the NR, KOG, KEGG, Swiss-Prot, and GO databases. There were 6181 (66.31%) annotated genes for the 3 main GO categories of biological process, cellular component, and molecular function, including 48 subcategories (Figure 3a). The molecular function component was mainly distributed across molecular activity, ion binding, and oxidoreductase activity. The biological processes, cellular nitrogen compound metabolic processes, and biosynthetic processes contained the most proteins. However, compared with SPS-2, the molecular function component of *A. alternata* Y784-BC03 was detected to be involved in catalysis, binding, and transport [24]. To further understand the functions of the strain SPS-2 protein, 3241 (34.77%) genes were annotated and assigned to 45 different KEGG pathways (Figure 3b). “Carbohydrate metabolism” was the most enriched pathway, followed by “amino acid metabolism” and “translation”. These results suggested the presence of an enriched and varied array of carbohydrates and amino metabolic functions that enable higher energy conversion efficiency. Similarly, the KEGG analysis of the predicted genes of strains Y784-BC03 revealed an abundant number of metabolic pathways, and many of the predicted genes were associated with the biosynthesis of SMs. Among these 25 KOG functional categories, most of the genes were associated with “carbohydrate transport and metabolism”, followed by “post-translational modification, protein”, “secondary metabolite biosynthesis, transport, and catabolism”, and “amino acid transport and metabolism” (Figure 3c).

### 3.4. CAZyme Analysis

**Figure 4:**
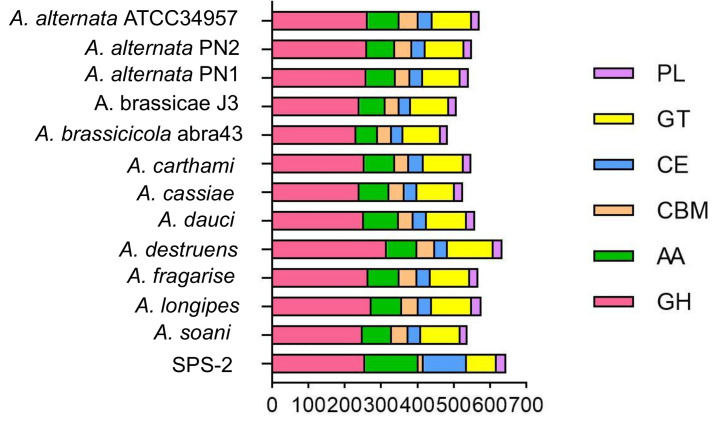
CAZyme family members of strain SPS-2 and other *Alternaria* strains deposited in the CAZy database.

CAZymes as one of the most important gene families in the fungal genome are involved in lignocellulose degradation and some other biological processes [25,26]. A total of 644 genes were annotated as the CAZyme family in strain SPS-2, including 257 glycoside hydrolases (GHs), 146 auxiliary activities (AA), 118 carbohydrate esterases (CEs), 83 glycosyl transferases (GTs), 25 polysaccharide lyases (PLs), and 15 carbohydrate-binding modules (CBMs) (Figure 4, Table S2). Accordingly, GHs occupied the predominant genes in all the predicted CAZymes of strain SPS-2. By comparison with other *Alternaria* strains from the CAZy database, it was found that this strain harbored the most abundant CAZymes including GHs, CEs, and PLs, suggesting that it has the strongest capability for plant biomass decomposition [27].

### 3.5. Analysis of Secondary Metabolite Biosynthetic Potential

**Figure 5:**
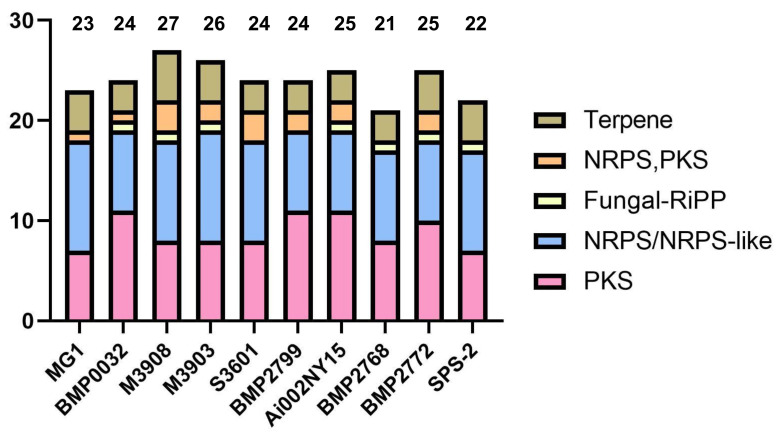
The number (*y*-axis) and type of secondary metabolite BGCs in strain SPS-2 and other *Alternaria* strains deposited in the antiSMASH database.

**Figure 6:**
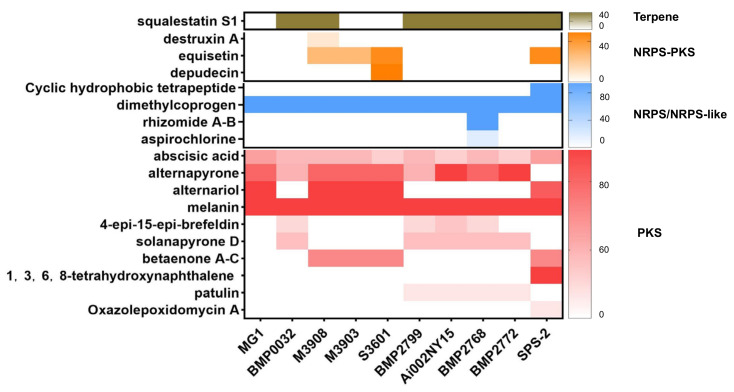
Putative secondary metabolites produced by strain SPS-2 and other *Alternaria* strains deposited in the antiSMASH database.

The antiSMASH results indicated that strain SPS-2 possessed 22 BGCs for SM biosynthesis, which was similar to the other 10 *Alternaria* strains (Figure 5, Table S3). Additionally, its BGC inventory consisted of 10 NRPSs, 7 PKSs, 4 terpenes, and 1 fungal-RiPP, of which 8 BGCs with high similarity with known gene clusters are putatively responsible for the production of equisetin, betaenones A–C, alternariol, dimethylcoprogen, and melanin (Figure 6, Table S4). However, the biosynthetic products of other BGCs in strain SPS-2 cannot be characterized and need to be further investigated and unveiled.

**Figure 7:**
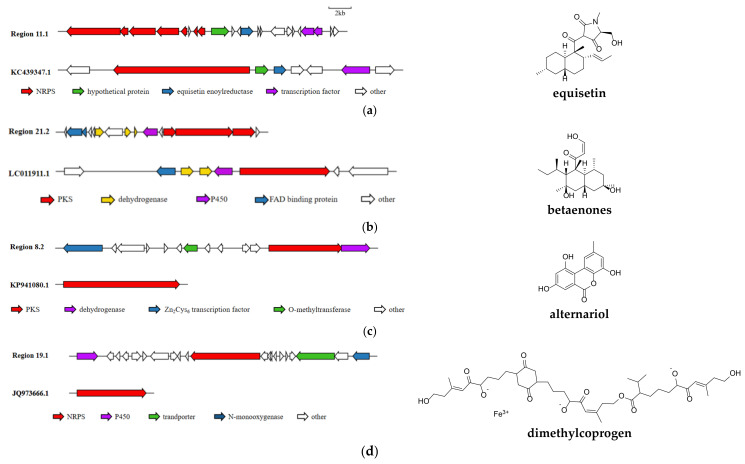

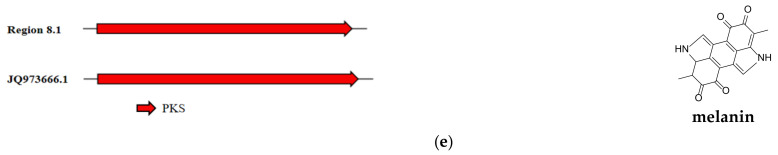
Five identified BGCs with high identity in strain SPS-2 responsible for biosynthesis of (**a**) equisetin, (**b**) betaenones, (**c**) alternariol, (**d**) dimethylcoprogen, and (**e**) melanin.

Region 11.1, one NRPS BGC, displayed 45% similarity with the BGC from *F. heterosporum* (GenBank: KC439347.1) responsible for the biosynthesis of equisetin, which is an antibacterial agent and selectively inhibits *Staphylococci* and *Mycobacteria* by inhibiting specific ATPases or ionophores in bacterial and mitochondrial inner membranes [28]. Although some genes of region 11.1 showed high similarity with equisetin synthetase (Figure 7a, Table S5) [29], other genes did not share significant sequence homology, suggesting that region 11.1 might produce other alkaloids with similar structures of equisetin.

Region 21.2 displayed significant similarity with the BGC of betaenones A–C in *Phoma betae* (GenBank: LC011911.1). These metabolites exhibited strong inhibitory effects on PKC, CDK4, and EGF-R tyrosine kinases, with the corresponding IC_50_ values of 36.0, 11.5, and 10.5 µM, respectively, and antiangiogenic activity [30]. Genes *Bet1* and *Bet3* with similarities of 80% and 90%, respectively, are key enzymes in betaenone biosynthesis (Figure 7b, Table S6) [31].

Region 8.2 displayed a high similarity with alternariol (AOH) BGC from *Parastagonospora nodorum* SN15 (GenBank: KP941080.1). Core gene *ctg*_*1223* is a PKS-related enzyme, which had a significant BLAST hit with the key gene AKN45693.1 for the synthesis of AOH, an important mycotoxin used to alter the action of glutathione and the enzymes involved in the redox system as well as causing DNA damage [32]. Gene *pksI* is a core gene for AOH synthesis, and it usually co-expresses with other genes including O-methyltransferases, FAD-dependent monooxygenases, short-chain dehydrogenase, and other different enzymes to produce alternariol derivatives (Figure 7c, Table S7) [33].

One PKS BGC with 100% homology to that (GenBank: JQ973666.1) of *A. alternate* presumably for dimethylcoprogen biosynthesis was discovered in region 19.1. This compound is a novel trihydroxamate siderophore [34], and its core NRPS gene was found to have a highly similar sequence to that of AFN69082.1 in BGC0001249 (Figure 7d, Table S8).

Region 8.1 displayed 100% similarity with the BGC (GenBank: JQ973666.1) responsible for the biosynthesis of melanin, which is a ubiquitous pigment with potent resistance to environmental stress such as UV radiation [35]. Its *pksI* was determined as the essential gene for melanin biosynthesis in *Bipolaris oryzae* (Figure 7e, Table S9) [36].

### 3.6. Molecular Networking Analysis

**Figure 8:**
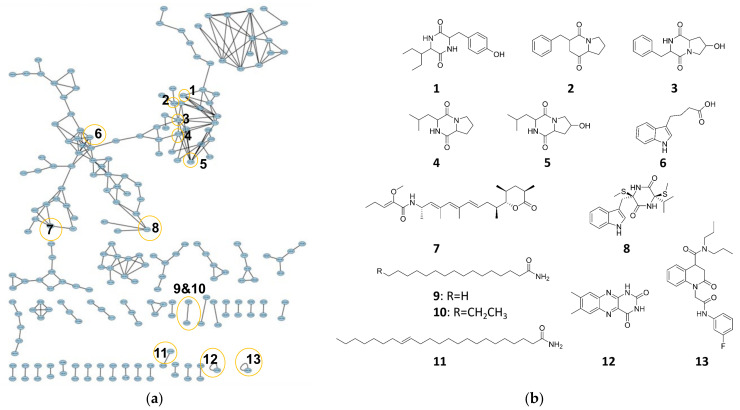
Metabolomic analysis of strain SPS-2: (**a**) molecular network (each node represents parent ion of each SM); (**b**) putative SMs of strain SPS-2 annotated by GNPS.

The feature-based molecular networking for the metabolite profile of strain SPS-2 was created with the Global Natural Products Social Molecular Networking (GNPS) analysis tool. In addition to five cyclodipeptides (**1**–**5**), a number of alkaloids (**6**–**13**) were detected and characterized in the crude extract of strain SPS-2 (Figure 8). Among these SMs, compound **7** was originally obtained from strain *Sorangium cellulosum*. Therefore, ce38 exhibited potent anticancer activity and a strong effect on lysosomes [37]. Compound **11** displayed a cytotoxic effect on HepG-2 cells and had the potential to antagonize depression and anxiety [38,39], while compound **12** had AChE inhibitory capacity, with an IC_50_ value of 12.24 ± 0.12 [40], and **13** showed an antiviral effect [41]. These results indicated strain SPS-2 has great potential to biosynthesize many bioactive SMs. However, the GNPS-predicted compounds were not similar to those analyzed with antiSMASH, which might be attributed to the fact that some BGCs in strain SPS-2 are silent under traditional laboratory conditions. Furthermore, substances with molecular weights of over 600 Dalton cannot be identified in the GNPS repository, suggesting that this strain has the potential to produce new SMs.

## 4. Conclusions

In this work, a high-quality *de novo* genome of an endophytic *Alternaria* sp. SPS-2 was obtained via whole-genome sequencing and assembly. Genome analysis suggested that strain SPS-2 is capable of encoding a wide array of CAZy and secondary metabolic enzymes. The antiSMASH results indicated that this strain harbored 22 secondary metabolite BGCs including 7 PKSs, 10 NRPSs, 4 Terpenes, and 1 fungal-RiPP, and 14 of these BGCs are unknown and have the biosynthetic potential of new SMs. LS–MS/MS and GNPS-based analyses suggested that this endophytic fungus is a potential producer of bioactive SMs. Therefore, future efforts should be focused on awakening these cryptic BGCs in strain SPS-2 to produce more SMs using various approaches, such as OSMAC strategy [42], heterologous gene expression [43], and transcriptional regulation [44].

## Figures and Tables

**Table 1:** General genomic features of strain SPS-2.

Item	Value
Total length	33,400,178
Total sequence number	38
Max length	4,684,119
GC content	51.0%
N50	2,610,814
Total genes length	14,826,936
Total genes number	9789
Total CDSs length (bp)	13,553,185
CDSs percentage of genome	40.58%

**Table 2:** Genomic features of all *Alternaria* strains available in the NCBI database.

Strain	Genome Size (Mb)	GC%	Scaffolds	N50 (Mb)	Genes	Sequencing Technology	Assembly ID
*A. arborescens* FERA675	33.94	51.10	325	-	12,923	Illumina MiSeq	GCA_004154835.1
*A. rosae* MPI-PUGE-AT-0040	33.83	52	52	3.28	12,727	PacBio SEQUEL	GCA_020736505.1
*A. longipes* CBS 540.94	39.43	50.99	15	3.60	-	Oxford Nanopore	GCA_019059555.1
*A. burnsii* CBS107.38	32.96	50.9	67	1.49	11,314	Illumina HiSeq	GCA_013036055.1
*A. tenuissima* FERA1166	35.7	51.1	22	2.58	13,575	Illumina MiSeq	GCA_004156035.1
*A. alternata* SRC1lrK2f	32.99	51.4	79	1.1	13,577	Illumina	GCA_001642055.1
*A. ethzedia* BMP 0044	36.02	50.4	579	0.86	11,096	Illumina HiSeq	GCA_023757985.1
BMP 2799	36.4	50.2	225	1.0	11,159	Illumina HiSeq	GCA_023758145.1
*A. alternata* EV-MIL-31	34.96	51	61	2.28	24,347	Illumina MiSeq	GCA_016097525.1
*A. hordeiaustralica* BMP2776	36.96	49.9	188	0.95	11,145	Illumina HiSeq	GCA_023758085.1
*A. novae-zelandiae* BMP2774	37.57	49.5	204	1.24	11,027	Illumina HiSeq	GCA_023758075.1
*A. ventricosa* BMP2768	34.69	51.4	89	1.33	11,061	Illumina HiSeq	GCA_023758065.1
*A. triticimaculans* BMP 0046	36.79	49.9	236	1.67	11,104	Illumina HiSeq	GCA_023758025.1
*A. viburni* BMP2772	36.35	50.3	188	1.05	11,586	Illumina HiSeq	GCA_023758015.1
BMP0032	37.29	49.7	196	0.98	11,103	Illumina HiSeq	GCA_023758005.1
*A. panax* BNCC115425	34.76	51	24	2.78	11,838	Illumina HiSeq; PacBio	GCA_019702505.1
M3908	33.77	51.1	118	0.77	-	Illumina HiSeq	GCA_023621335.1
M3903	33.77	51.1	104	0.95	-	Illumina HiSeq	GCA_023621305.1
S3601	34.24	50.9	75	1.30	-	Illumina HiSeq	GCA_023621325.1
MG1	34.7	49.9	130	1.66	13,440	Illumina	GCA_003574525.1
SPS-2	33.4	51	38	2.61	9789	Illumina MiSeq	JAMXIG000000000

“-“indicated unknown.

**Table 3:** Genomic assembly and functional annotation of strain SPS-2.

Type	Repeat Number	Repeat Size	In Genome (%)
rRNA	12	6641	0.0199
tRNA	108	9657	0.0289
ncRNA	32	4218	0.0126
LTR	19	26,157	0.0008
DNA	29	12,782	0.0004

## Data Availability

The complete genome sequence data reported in this study have been deposited at GenBank under accession no.117004 (BioProject: PRJNA832572).

## Supplementary Materials

The following supporting information can be downloaded at: https://www.mdpi.com/article/10.3390/microorganisms10091789/s1, Table S1: Genome assembly integrity assessment for strain SPS-2; Table S2: CAZyme profiles of strain SPS-2 and other twelve *Alternaria* strains deposited in CAZyme databases; Table S3: The number and type of secondary metabolite BGCs in strain SPS-2 and other *Alternaria* strains deposited in antiSMASH database; Table S4: Secondary metabolite BGCs of strain SPS-2 by antiSMASH analysis; Table S5: BGC component for equisetin in strain SPS-2; Table S6: BGC component for betaenones in strain SPS-2; Table S7: BGC component for alternariol in strain SPS-2; Table S8: BGC component for dimethylcoprogen in strain SPS-2; Table S9: BGC component for melanin in strain SPS-2.

## References

[1] Gunatilaka A.A.L. (2006). Natural products from plant-associated microorganisms: Distribution, structural diversity, bioactivity, and implications of their occurrence. J. Nat. Prod..

[2] Hridoy M., Gorapi M.Z.H., Noor S., Chowdhury N.S., Rahman M.M., Muscari I., Masia F., Adorisio S., Delfino D.V., Mazid M.A. (2022). Putative anticancer compounds from plant-derived endophytic fungi: A review. Molecules.

[3] Lou J., Fu L., Peng Y., Zhou L. (2013). Metabolites from *Alternaria* fungi and their bioactivities. Molecules.

[4] Su-shi L., Qian L., Jie-yu Z., Li L., Tao W. (2021). Research progress on active secondary metabolites from *Alternaria*. Nat. Prod. Res. Dev..

[5] Kong F.D., Yi T.F., Ma Q.Y., Xie Q.Y., Zhou L.M., Chen J.P., Dai H.F., Wu Y.G., Zhao Y.X. (2020). Biphenyl metabolites from the patchouli endophytic fungus *Alternaria* sp. PfuH1. Fitoterapia.

[6] Zhang H.W., Ruan C.F., Bai X.L. (2015). Isolation and antimicrobial effects of endophytic fungi from *Edgeworthia chrysantha*. Bangl. J. Pharmacol..

[7] Wang S., Cheng Y. (2006). Separation and determination of the effective components in the alabastrum of *Edgeworthia chrysantha* Lindl. by micellar electrokinetic capillary chromatography. J. Pharm. Biomed. Anal..

[8] Jiang L., Lim C.J., Jeong J.C., Kim C.Y., Kim D.-H., Kim S.W., Lee J. (2019). Whole-genome sequence data and analysis of *Saccharibacillus* sp. ATSA2 isolated from Kimchi cabbage seeds. Data Brief.

[9] Schubert M., Lindgreen S., Orlando L. (2016). AdapterRemoval v2: Rapid adapter trimming, identification, and read merging. BMC Res. Notes.

[10] Walker B.J., Abeel T., Shea T., Priest M., Abouelliel A., Sakthikumar S., Cuomo C.A., Zeng Q., Wortman J., Young S.K. (2014). Pilon: An integrated tool for comprehensive microbial variant detection and genome assembly improvement. PLoS ONE.

[11] Waterhouse R.M., Seppey M., Simao F.A., Manni M., Ioannidis P., Klioutchnikov G., Kriventseva E.V., Zdobnov E.M. (2018). BUSCO applications from quality assessments to gene prediction and phylogenomics. Mol. Biol. Evol..

[12] Korf I. (2004). Gene finding in novel genomes. BMC Bioinf..

[13] Stanke M., Morgenstern B. (2005). AUGUSTUS: A web server for gene prediction in eukaryotes that allows user-defined constraints. Nucleic Acids Res..

[14] Majoros W.H., Pertea M., Salzberg S.L. (2004). TigrScan and GlimmerHMM: Two open source ab initio eukaryotic gene-finders. Bioinformatics.

[15] Lowe T.M., Eddy S.R.J.N.a.r. (1997). tRNAscan-SE: A program for improved detection of transfer RNA genes in genomic sequence. Nucleic Acids Res..

[16] Lagesen K., Hallin P., Rodland E.A., Staerfeldt H.H., Rognes T., Ussery D.W. (2007). RNAmmer: Consistent and rapid annotation of ribosomal RNA genes. Nucleic Acids Res..

[17] Griffiths-Jones S., Moxon S., Marshall M., Khanna A., Eddy S.R., Bateman A. (2005). Rfam: Annotating non-coding RNAs in complete genomes. Nucleic Acids Res..

[18] Moriya Y., Itoh M., Okuda S., Yoshizawa A.C., Kanehisa M. (2007). KAAS: An automatic genome annotation and pathway reconstruction server. Nucleic Acids Res..

[19] Sangrador-Vegas A., Mitchell A.L., Chang H.Y., Yong S.Y., Finn R.D. (2016). GO annotation in InterPro: Why stability does not indicate accuracy in a sea of changing annotations. Database.

[20] Buchfink B., Xie C., Huson D.H.J.N.m. (2015). Fast and sensitive protein alignment using DIAMOND. Nat. Methods.

[21] Wang M., Carver J.J., Phelan V.V., Sanchez L.M., Garg N., Peng Y., Nguyen D.D., Watrous J., Kapono C.A., Luzzatto-Knaan T. (2016). Sharing and community curation of mass spectrometry data with Global Natural Products Social Molecular Networking. Nat. Biotechnol..

[22] Shannon P., Markiel A., Ozier O., Baliga N.S., Wang J.T., Ramage D., Amin N., Schwikowski B., Ideker T. (2003). Cytoscape: A software environment for integrated models of biomolecular interaction networks. Genome Res..

[23] Woudenberg J.H., Seidl M.F., Groenewald J.Z., de Vries M., Stielow J.B., Thomma B.P., Crous P.W. (2015). *Alternaria* section *Alternaria*: Species, formae speciales or pathotypes?. Stud. Mycol..

[24] Huang K., Tang J., Zou Y., Sun X., Lan J., Wang W., Xu P., Wu X., Ma R., Wang Q. (2021). Whole genome sequence of *Alternaria alternata*, the causal agent of black spot of Kiwifruit. Front. Microbiol..

[25] Pallister E., Gray C.J., Flitsch S.L. (2020). Enzyme promiscuity of carbohydrate active enzymes and their applications in biocatalysis. Curr. Opin. Struct. Biol..

[26] Garron M.L., Henrissat B. (2019). The continuing expansion of CAZymes and their families. Curr. Opin. Chem. Biol..

[27] Rajarammohan S., Paritosh K., Pental D., Kaur J. (2019). Comparative genomics of *Alternaria* species provides insights into the pathogenic lifestyle of *Alternaria brassicae*—a pathogen of the Brassicaceae family. BMC Genom..

[28] Hellwig V., Grothe T., Mayer-Bartschmid A., Endermann R., Geschke F.U., Henkel T., Stadler M. (2002). Altersetin, a new antibiotic from cultures of endophytic *Alternaria* spp. taxonomy, fermentation, isolation, structure elucidation and biological activities. J. Antibiot..

[29] Kakule T.B., Sardar D., Lin Z., Schmidt E.W. (2013). Two related pyrrolidinedione synthetase loci in *Fusarium heterosporum* ATCC 74349 produce divergent metabolites. ACS Chem. Biol..

[30] Brauers G., Edrada R.A., Ebel R., Proksch P., Wray V., Berg A., Grafe U., Schachtele C., Totzke F., Finkenzeller G. (2000). Anthraquinones and betaenone derivatives from the sponge-associated fungus *Microsphaeropsis* species: Novel inhibitors of protein kinases. J. Nat. Prod..

[31] Ugai T., Minami A., Fujii R., Tanaka M., Oguri H., Gomi K., Oikawa H. (2015). Heterologous expression of highly reducing polyketide synthase involved in betaenone biosynthesis. Chem. Commun..

[32] Chen L.-H., Lin C.-H., Chung K.-R. (2013). A nonribosomal peptide synthetase mediates siderophore production and virulence in the citrus fungal pathogen *Alternaria alternata*. Mol. Plant Pathol..

[33] Wenderoth M., Garganese F., Schmidt-Heydt M., Soukup S.T., Ippolito A., Sanzani S.M., Fischer R. (2019). Alternariol as virulence and colonization factor of *Alternaria alternata* during plant infection. Mol. Microbiol..

[34] Jalal M.A.F., Love S.K., van der Helm D. (1988). Nα-Dimethylcoprogens Three novel trihydroxamate siderophores from pathogenic fungi. Biol. Met..

[35] Kihara J., Moriwaki A., Ueno M., Tokunaga T., Arase S., Honda Y. (2004). Cloning, functional analysis and expression of a scytalone dehydratase gene (SCD1) involved in melanin biosynthesis of the phytopathogenic fungus *Bipolaris oryzae*. Curr. Genet..

[36] Moriwaki A., Kihara J., Kobayashi T., Tokunaga T., Arase S., Honda Y. (2004). Insertional mutagenesis and characterization of a polyketide synthase gene (PKS1) required for melanin biosynthesis in *Bipolaris oryzae*. FEMS Microbiol. Lett..

[37] Jahns C., Hoffmann T., Muller S., Gerth K., Washausen P., Hofle G., Reichenbach H., Kalesse M., Muller R. (2012). Pellasoren: Structure elucidation, biosynthesis, and total synthesis of a cytotoxic secondary metabolite from *Sorangium cellulosum*. Angew. Chem. Int. Ed. Engl..

[38] Zhu Y., Han S., Li J., Gao H., Dong B. (2022). Aqueous extract of sea squirt (*Halocynthia roretzi*) with potent activity against human cancer cells acts synergistically with doxorubicin. Mar. Drugs.

[39] Li M.M., Jiang Z.E., Song L.Y., Quan Z.S., Yu H.L. (2017). Antidepressant and anxiolytic-like behavioral effects of erucamide, a bioactive fatty acid amide, involving the hypothalamus-pituitary-adrenal axis in mice. Neurosci. Lett..

[40] Alves A.J.S., Pereira J.A., Dethoup T., Cravo S., Mistry S., Silva A.M.S., Pinto M.M.M., Kijjoa A. (2019). A new meroterpene, a new benzofuran derivative and other constituents from cultures of the marine sponge-associated fungus *Acremonium persicinum* KUFA 1007 and their anticholinesterase activities. Mar. Drugs.

[41] El-Helw E.A.E., Morsy A.R.I., Hashem A.I. (2021). Evaluation of some new heterocycles bearing2-oxoquinolylmoiety as immunomodulator against highly pathogenic avian influenza virus (H5N8). J. Heterocycl. Chem..

[42] Pan R., Bai X., Chen J., Zhang H., Wang H. (2019). Exploring structural diversity of microbe secondary metabolites using OSMAC strategy: A literature review. Front. Microbiol..

[43] Xu Y., Du X., Yu X., Jiang Q., Zheng K., Xu J., Wang P. (2022). Recent advances in the heterologous expression of biosynthetic gene clusters for marine natural products. Mar. Drugs.

[44] Yin W., Keller N.P. (2011). Transcriptional regulatory elements in fungal secondary metabolism. J. Microbiol..

