# Supplementary material for: Whole-Genome Sequence Analysis of an Endophytic Fungus Alternaria sp. SPS-2 and Its Biosynthetic Potential of Bioactive Secondary Metabolites
Source: Microorganisms. 2022 Sep 5;10(9):1789. doi: 10.3390/microorganisms10091789 (PMC9503250; doi:10.3390/microorganisms10091789)
Supplement: Supplementary file 1 [file microorganisms-10-01789-s001.zip › microorganisms-1851226-supplementary.pdf]

Supplementary materials for

Whole-genome sequence analysis of an endophytic fungus *Alternaria* sp. SPS-2 and its biosynthetic potential of bioactive secondary metabolites

Jianyun Tao, Xuelian Bai, Mingyuan Zeng, Mengshi Li, Zhe Hu, Yunfen Hua* and Huawei Zhang*

* Correspondence: huayf@zjut.edu.cn (Y.H.); hwzhang@zjut.edu.cn (H.Z.); Tel.: +86-571-8832091

Contents

Table S1. Genome assembly integrity assessment for strain SPS-2----------2
Table S2. CAZyme profiles of strain SPS-2 and other twelve *Alternaria* strains deposited in CAZyme database----------3
Table S3. The number and type of secondary metabolite BGCs in strain SPS-2 and other selective *Alternaria* strains deposited in NCBI database----------4
Table S4. Secondary metabolite BGCs of strain SPS-2 by antiSMASH analysis ----------5
Table S5. BGC component for equisetin in strain SPS-2----------6
Table S6. BGC component for betaenones in strain SPS-2----------7
Table S7. BGC component for alternariol in strain SPS-2 ----------8
Table S8. BGC component for dimethylcoprogen in strain SPS-2----------9
Table S9. BGC component for melanin in strain SPS-2----------10

Table S1. Genome assembly integrity assessment for strain SPS-2.

Property	Number	Percentage (%)
Complete BUSCO	276	95.2
Missing genes	10	3.4
Total BUSCO group searched	290	100

Table S2. CAZyme profiles of strain SPS-2 and other twelve *Alternaria* strains deposited in CAZyme database.

Strain	CAZy fimily						Total
	GH	CBM	CE	AA	GT	PL	
Alternaria solani	249	46	36	80	107	20	538
A. longipes	273	45	37	85	111	25	576
A. fragariae	265	48	37	86	109	23	568
A. destrueens	316	48	36	84	125	26	635
A. dauci	252	40	37	97	111	22	559
A. cassiae	240	42	34	83	104	22	525
A. carthami	255	39	40	83	110	22	549
A. brassicicola abra43	231	38	32	60	103	20	484
A. brassicae J3	241	38	33	71	104	21	508
A. alternaata PN1	260	41	35	80	103	23	542
A. alternaata PN1	261	48	36	77	107	21	550
A. alternaata ATCC34957	264	53	37	87	109	21	622
SPS-2	257	15	118	146	83	25	644

Table S3. The number and type of secondary metabolite BGCs in strain SPS-2 and other selective *Alternaria* strains deposited in NCBI database.

BGC Type	Strain									
	MG1	BMP0032	M3908	M3903	S3601	BMP2799	Ai002NY15	BMP2768	BMP2772	SPS-2
PKS	7	11	8	8	8	11	11	8	10	7
NRPS/NRPS-like	11	8	10	11	10	8	8	9	8	10
Fungal-RiPP	-	1	1	1	-	-	1	1	1	1
NRPS, PKS	1	1	3	2	3	2	2	-	2	0
Terpene	4	3	5	4	3	3	3	3	4	4

Table S4. Secondary metabolite BGCs of strain SPS-2 by antiSMASH analysis.

Gene cluster	Type	Gene cluster position	predicted product (similarity)
Region 1.1	NRPS	3405290-3461377	Unknown
Region 2.1	Terpene	1004114-1024548	Unknown
Region 3.1	T3PKS	985697-1026775	Unknown
Region 4.1	Terpene	388980-408695	Squalestatin S1 (40%)
Region 4.2	Terpene	2345955-2367289	Unknown
Region 5.1	T1PKS	1087051-1131054	Oxazolepoxidomycin A (13%)
Region 5.2	T1PKS	2115332-2162885	Abscisic acid (50%)
Region 6.1	T1PKS	252626-300167	Equisetin (18%)
Region 7.1	Fungal-RiPP	280898-318613	Unknown
Region 7.2	NRPS	1415890-1477928	Cyclic hydrophobic tetrapeptide (100%)
Region 8.1	T1PKS	160848-205614	Melanin (100%)
Region 8.2	T1PKS	918158-961487	1,3,6,8-tetrahydroxynaphthalene (100%)
Region 8.3	NRPS	1667861-1723346	Unknown
Region 9.1	NRPS, indole	74519-134677	Unknown
Region 9.2	Terpene	1083778-1105346	Unknown
Region 9.3	NRPS-like	1628313-1671303	Unknown
Region 11.1	NRPS	383909-428735	Equisetin (45%)
Region 12.1	NRPS-like	821562-865410	Unknown
Region 17.1	NRPS-like	305087-348950	Unknown
Region 19.1	NRPS	260997-319962	Dimethylcoprogen (100%)
Region 21.1	NRPS-like	36305-79148	Unknown
Region 21.2	T1PKS	174713-214266	Betaenone (62%)

Table S5. BGC component for equisetin in strain SPS-2.

Gene location	Description	MIBiG cluster	Identity	Coverage
ctg11_494	equisetin synthetase	BGC0001255	55%	100.4%
ctg11_495	equisetin synthetase	BGC0001255	76%	93.6%
ctg11_496	equisetin synthetase	BGC0001255	56%	101.0%
ctg11_497	equisetin synthetase	BGC0001255	65%	100.5%
ctg11_498	equisetin synthetase	BGC0001255	65%	100.0%
ctg11_500	equisetin synthetase	BGC0001255	89%	99.1%
ctg11_501	equisetin synthetase	BGC0001255	78%	98.6%
ctg11_502	hypothetical protein	BGC0001255	55%	69.9%
ctg11_505	equisetin enoylreductase	BGC0001255	70%	100.0%
ctg11_508	C6_transcription_factor	BGC0001738	21%	109.6%
ctg11_512	putative fungal transcription factor	BGC0001255	60%	99.7%
ctg11_513	putative fungal transcription factor	BGC0001255	47%	39.5%
ctg11_515	putative MFS transporter	BGC0001255	81%	81.7%

Table S6. BGC component for betaenones in strain SPS-2.

Gene location	Description	MIBiG cluster	Identity	Coverage
ctg21_238	putative FAD binding domain protein	BGC0001280	78%	97.8%
ctg21_239	putative FAD binding domain protein	BGC0001280	76%	62.8%
ctg21_242	short-chain dehydrogenase	BGC0001280	85%	87.1%
ctg21_243	P450_monooxygenase	BGC0000818	36%	99.0%
ctg21_244	zinc-binding dehydrogenase family protein	BGC0001280	87%	100.0%
ctg21_246	cytochrome_P450	BGC0001280	90%	79.6%
ctg21_248	polyketide synthase	BGC0001280	88%	81.1%
ctg21_249	polyketide synthase	BGC0001280	81%	98.7%
ctg21_250	polyketide synthase	BGC0001280	85%	99.7%
ctg21_251	MFS transporter, putative	BGC0001280	52%	79.5%

Table S7. BGC component for alternariol in strain SPS-2.

Gene location	Description	MIBiG cluster	Identity	Coverage
ctg8_1212	PtaR3	BGC0000121	26%	73.3%
ctg8_1214	monooxygenase, FAD binding domain cpaO	BGC0001563	36%	85.4%
ctg8_1218	o-methyltransferase B	BGC0000004	40%	73.5%
ctg8_1221	polyketide synthase	BGC0001284	85%	100.0%
ctg8_1222	polyketide synthase	BGC0001284	68%	90.4%
ctg8_1223	polyketide synthase	BGC0001284	85%	98.9%
ctg8_1224	polyketide synthase	BGC0001284	84%	96.3%

Table S8. BGC component for dimethylcoprogen in strain SPS-2.

Gene location	Description	MIBiG cluster	Identity	Coverage
ctg19_345	Bcaba1	BGC00001893	41%	88.3%
ctg19_355	nonribosomal_peptide_synthetase_6	BGC0001249	98%	99.8%
ctg19_363	SirA	BGC0001044	36%	96.4%
ctg19_364	multidrug resistance protein	BGC0000337	26%	85.8%
ctg19_365	lysine N(6)-hydroxylase/L-ornithine-N(5)-oxygenase family protein	BGC0002001	29%	73.8%

Table S9. BGC component for melanin in strain SPS-2.

Gene location	Description	MIBiG cluster	Identity	Coverage
ctg8_253	polyketide synthase	BGC00001265	88%	99.5%
